# Volume-based referral for cardiovascular procedures in the United States: a cross-sectional regression analysis

**DOI:** 10.1186/1472-6963-5-42

**Published:** 2005-06-03

**Authors:** Andrew J Epstein, Saif S Rathore, Harlan M Krumholz, Kevin GM Volpp

**Affiliations:** 1 Division of Health Policy and Administration, Department of Epidemiology and Public Health, Yale University School of Medicine, New Haven, Connecticut, USA; 2 Section of Cardiovascular Medicine, Department of Internal Medicine, Yale University School of Medicine, New Haven, Connecticut, USA; 3 Yale-New Haven Hospital Center for Outcomes Research and Evaluation, New Haven, Connecticut, USA; 4 Center for Health Equity Research and Promotion, Philadelphia Veterans' Affairs Hospital, Philadelphia, Pennsylvania, USA; 5 Department of Health Care Systems, Wharton School of Business, and Section of General Internal Medicine, Department of Medicine, School of Medicine, both at University of Pennsylvania, Philadelphia, Pennsylvania, USA

## Abstract

**Background:**

We sought to estimate the numbers of patients affected and deaths avoided by adopting the Leapfrog Group's recommended hospital procedure volume minimums for coronary artery bypass graft (CABG) surgery and percutaneous coronary intervention (PCI). In addition to hospital risk-adjusted mortality standards, the Leapfrog Group recommends annual hospital procedure minimums of 450 for CABG and 400 for PCI to reduce procedure-associated mortality.

**Methods:**

We conducted a retrospective analysis of a national hospital discharge database to evaluate in-hospital mortality among patients who underwent PCI (n = 2,500,796) or CABG (n = 1,496,937) between 1998 and 2001. We calculated the number of patients treated at low volume hospitals and simulated the number of deaths potentially averted by moving all patients to high volume hospitals under best-case conditions (i.e., assuming the full volume-associated reduction in mortality and the capacity to move all patients to high volume hospitals with no related harms).

**Results:**

Multivariate adjusted odds of in-hospital mortality were higher for patients treated in low volume hospitals compared with high volume hospitals for CABG (OR 1.16, 95% CI 1.10–1.24) and PCI (OR 1.12, 95% CI 1.05–1.20). A policy of hospital volume minimums would have required moving 143,687 patients for CABG and 87,661 patients for PCI from low volume to high volume hospitals annually and prevented an estimated 619 CABG deaths and 109 PCI deaths. Thus, preventing a single death would have required moving 232 CABG patients or 805 PCI patients from low volume to high volume hospitals.

**Conclusion:**

Recommended hospital CABG and PCI volume minimums would prevent 728 deaths annually in the United States, fewer than previously estimated. It is unclear whether a policy requiring the movement of large numbers of patients to avoid relatively few deaths is feasible or effective.

## Background

Patients treated at hospitals with higher volumes of cardiovascular procedures, including coronary artery bypass graft (CABG) surgery and percutaneous coronary intervention (PCI), are reported to have better outcomes than patients treated at hospitals with lower volumes [1-3]. These data have led to a growing interest in using volume to characterize hospital quality of care for cardiovascular procedures by purchaser and consumer organizations [4,5]. As part of its "Evidence-Based Hospital Referral" guidelines, the Leapfrog Group, a coalition of large health care purchasers that insure 34 million Americans, recommended until 2003 that its members contract for selected procedures, including CABG and PCI, only with hospitals that met minimum volume thresholds [6]. Hospital risk-adjusted mortality criteria were added to the Leapfrog guidelines in 2003 [7].

Although proponents contend that implementing volume-based thresholds will reduce procedure-associated mortality, there are limited data on the number of patients affected by the adoption of volume minimums and the magnitude of any potential benefits. One study of patients hospitalized in California in 1997 suggested that 338 deaths might be prevented in that state each year by adopting volume minimums for CABG and PCI [8], and another study estimated that applying hospital volume minimums for CABG and PCI nationwide would save 1,871 lives annually [9]. However, both studies relied on estimates of hospital CABG [1] and PCI [3] volume-mortality associations using New York state data from the late 1980s and early 1990s. Recent research by Birkmeyer and Dimick [7] uses data from 2000 to estimate the volume-mortality association and the national impact of the Leapfrog volume standards, and finds that 148,508 CABG cases would have to be moved to avert 594 deaths and 91,153 PCI cases would have to be moved to avert 547 deaths.

Because many purchasers have begun selective referral to providers that meet these Leapfrog criteria, it is important to understand the potential benefits and costs of the Leapfrog quality improvement recommendations. To provide an alternative, generalizable forecast of the potential consequences of adopting a volume-based referral policy, we conducted an evaluation of a hypothetical nationwide implementation of the Leapfrog Group's volume-based standards, the only criteria in effect until 2003, for CABG and PCI using nationally representative data from 1998–2001. We specifically sought to estimate the number of patients at low volume hospitals who would need to be moved to high volume hospitals and the number of deaths potentially averted by the adoption of hospital volume minimums.

## Methods

### National Inpatient Sample

Our analysis was based on the Nationwide Inpatient Sample (NIS), a hospital discharge database from the Agency for Healthcare Research and Quality's Health Care Utilization Project [10]. As the largest publicly available all-payer inpatient database in the United States, the NIS contains administrative records for all hospitalizations in a randomly selected national sample of non-governmental, acute care hospitals. The 2001 NIS, the most recent version of the NIS available at the time of our study, contains information on more than 7.4 million discharges from nearly 1,000 hospitals in 33 states, corresponding to nearly 20% of all admissions to US non-federal hospitals [11]. The NIS contains de-identified, hospitalization-level data, including information on primary and secondary diagnoses, demographic characteristics, procedure use, length of stay, payer, total charges, and admission and discharge status. Our study pooled data from the 1998, 1999, 2000, and 2001 NIS releases.

### Study sample

We created separate, procedure-specific cohorts for hospitalizations in which a patient had any procedure code indicating a CABG (International Classification of Diseases, 9th Edition Clinical Modification [ICD-9-CM] codes 36.10–36.2) or PCI (ICD-9-CM codes 36.00–36.06 and 36.09). Of the nearly 29 million records in the 1998, 1999, 2000, and 2001 NIS, we identified 306,942 hospitalizations with a CABG and 517,178 hospitalizations with a PCI. We excluded patients under the age of 18 and neonatal or obstetric admissions in order to restrict our evaluation to a typical adult population. Hospitalizations with missing data for sex, age, or mortality were also excluded. To limit administrative data coding errors, we excluded patients treated at hospitals with fewer than 10 CABGs in any year from the CABG cohort and patients treated at hospitals with fewer than 5 PCIs in any year from the PCI cohort. Finally, following the Leapfrog Group's policy recommendation [12], we restricted our analysis to admissions at hospitals located in US Census Bureau-defined Metropolitan Statistical Areas, which are clusters of counties comprising large population centers. The two final unweighted procedure cohorts consisted of 296,135 hospitalizations for CABG drawn from 746 hospital-years of data and 496,252 hospitalizations for PCI drawn from 851 hospital-years of data. With the appropriate NIS sampling weights, these data represented 1,496,937 hospitalizations for CABG from 3,365 hospital-years and 2,500,796 hospitalizations for PCI from 4,141 hospital-years.

### Hospital volume groups

To assess the association of hospital CABG and PCI volume and patient mortality, hospitals were divided into separate groups based on their annual volume. Hospitals were categorized as low volume if their annual volume was below the procedure-specific volume minimum recommended by the Leapfrog Group (450 cases for CABG, 400 cases for PCI) [7]. All other hospitals were considered to be high volume.

### Statistical analysis

Patient characteristics, including demographics, admission type, comorbidities, and payer, were compared between patients treated in low volume hospitals and high volume hospitals within each procedure cohort using global chi-square analyses for categorical variables and simple t-tests for continuous variables.

The principal study outcome was in-hospital mortality. We compared crude rates of mortality between patients treated in high volume hospitals and low volume hospitals in each procedure cohort using global chi-square analysis. Unadjusted and multivariable logistic regressions that accounted for the NIS survey design were conducted within each procedure cohort to assess the association between treatment at a low volume hospital and patient mortality. Patient characteristics incorporated in the multivariable models, which were derived from previous administrative data-based evaluations of CABG and PCI and clinical judgment, included: sex, race (white, black, other), age (<65 years, 65–74 years, ≥75 years), year, admission source, urgency of admission (emergent, urgent, elective, unknown/missing), coronary artery disease (principal diagnosis of MI [ICD-9-CM code 410], secondary diagnosis of MI, any non-MI coronary disease diagnosis [ICD-9-CM codes 411–414], none), diabetes (ICD-9-CM code 250), chronic obstructive pulmonary disease (ICD-9-CM codes 490–496), hypertension (ICD-9-CM codes 401–405), renal dysfunction (ICD-9-CM codes 580–586), congestive heart failure (ICD-9-CM codes 428, 402.01, 402.11, 402.91, 404.01, 404.11, 404.91), and peripheral vascular disease (ICD-9-CM codes 440, 443).

In addition to factors common to both procedure models, we added selected covariates to specific procedure volume analyses. The multivariable model for CABG mortality accounted for concomitant valve repair and other open heart surgery procedures (ICD-9-CM procedure code 35), use of an internal mammary graft (ICD-9-CM procedure codes 36.15, 36.16), and a same admission PCI (ICD-9-CM procedure codes 36.00–36.06, 36.09). The analysis of hospital PCI volume controlled for multiple vessel PCI (ICD-9-CM procedure code 36.05).

### Estimating the effect of establishing hospital volume minimums

To assess the impact of hospital volume minimum policy for CABG and PCI, we calculated the average annual number of patients treated at low volume hospitals in each procedure cohort. Observations were weighted using NIS sampling weights to obtain nationally generalizable estimates.

We then estimated the number of deaths that could be prevented by the universal adoption of hospital volume minimums using mortality estimates obtained from the procedure-specific multivariable logistic regression models. Volume at low volume hospitals was modeled using a logarithmic transformation based on previous studies suggesting hospital volume-mortality associations exhibit a log-linear relationship [13-15]. Volume at high volume hospitals was modeled with a single dummy variable to reflect the average volume-associated mortality effect in high volume hospitals. An initial risk-adjusted probability of mortality was calculated for each hospitalization using the current distribution of patients across hospital volume groups. To derive a "best case" estimate of the impact of a hospital volume minimum policy, we assumed that all patients at low volume hospitals could be transported to high volume hospitals. A second risk-adjusted probability of mortality was then calculated for hospitalizations treated in low volume hospitals assuming that they had been treated in a typical high volume hospital by setting the hospital volume effect for low volume hospital patients to the average hospital volume effect for patients treated in high volume hospitals. This process removed any measured volume-associated difference in mortality between patients treated in low volume and high volume hospitals. Differences in weighted risk-adjusted probabilities between the two scenarios were summed across all low volume hospital patients to determine the estimated number of deaths averted by the adoption of hospital volume minimums for CABG and PCI.

Statistical analyses were conducted using SAS 8.2 (SAS Institute, Cary, NC) and Stata 8.2 (Stata Corporation, College Station, TX). Analysis of the NIS database was approved by the University of Pennsylvania Institutional Review Board.

## Results

### Patient characteristics

The proportion of patients treated at low volume hospitals was 14.0% for PCI and 38.4% for CABG. Mean patient age was 64.2 for PCI and 66.0 for CABG, and was generally comparable for patients treated in low volume and high volume hospitals. A greater proportion of patients treated at low volume CABG and PCI hospitals were non-white, while a lower proportion represented elective admissions or patients received in transfer as compared with patients treated at high volume hospitals for both procedures. The proportion of CABG patients receiving internal mammary artery grafts was slightly greater in high volume hospitals while the proportion of PCI patients with a myocardial infarction was slightly higher in low volume hospitals. Other patient characteristics, including sex distribution and prevalence of comorbid conditions, were generally comparable between patients at low volume and high volume hospitals (Table 1).

**Table 1:** Patient characteristics across hospital volume groups

**Characteristics**	**Hospital CABG Volume**				**Hospital PCI Volume**			
	**Overall**	**Low (<450)**	**High (≥450)**	**P**	**Overall**	**Low (<400)**	**High (≥400)**	**P**
% of patients (weighted)	100.0	38.4	61.6	-	100.0	14.0	86.0	-
% of hospital year groups*	100.0	69.8	30.2	-	100.0	45.1	54.9	-
								
Mean age, (SD) years	66.0 (0.07)	65.9 (0.08)	66.1 (0.11)	0.12	64.2 (0.08)	63.7 (0.12)	64.2 (0.09)	<0.001
Age				0.11				<0.001
Less than 65	41.1	41.7	40.7		48.7	50.7	48.3	
65–74 years	34.5	34.2	34.7		28.8	27.6	29.0	
75 years of age and older	24.4	24.1	24.6		22.5	21.7	22.6	
Male	69.5	69.4	69.5	0.90	65.5	64.4	65.7	<0.001
Race				0.014				0.016
White	64.2	59.2	67.3		63.3	56.7	64.4	
Black	3.8	3.8	3.9		4.5	5.1	4.4	
Other	7.5	9.5	6.2		7.3	10.1	6.9	
Race not reported/missing	24.5	27.6	22.6		24.9	28.1	24.4	
Primary payer				<0.001				<0.001
Medicare	53.8	51.9	55.0		49.0	45.7	49.5	
Medicaid	4.0	4.7	3.5		4.0	5.3	3.8	
Private	37.3	37.4	37.3		41.2	40.6	41.4	
Other/missing	4.9	6.0	4.2		5.8	8.4	5.4	
Diabetes	29.5	29.8	29.3	0.14	24.9	25.4	24.8	0.12
Hypertension	58.6	58.0	58.9	0.098	54.1	53.0	54.3	0.022
COPD	17.3	17.5	17.2	0.37	11.2	11.9	11.0	0.002
Congestive heart failure	17.6	17.5	17.7	0.64	9.9	10.7	9.8	<0.001
Peripheral vascular disease	7.6	7.5	7.6	0.55	5.6	5.4	5.6	0.43
Renal disease	6.0	5.8	6.1	0.11	2.6	2.9	2.5	<0.001
Coronary disease				<0.001				<0.001
MI as primary diagnosis	20.3	20.1	20.4		31.0	37.6	29.9	
MI as secondary diagnosis	5.7	6.3	5.3		4.5	5.0	4.4	
Other coronary artery disease	65.6	65.5	65.7		60.2	53.1	61.4	
No coronary disease	8.4	8.1	8.6		4.3	4.3	4.3	
Admission type				<0.001				<0.001
Emergency	23.7	23.8	23.6		31.9	36.9	31.1	
Urgent	25.2	22.1	27.1		26.1	23.6	26.5	
Elective	41.5	38.6	43.4		32.3	21.5	34.1	
Other/missing	9.6	15.6	5.9		9.6	18.1	8.2	
Arrived by inter-hospital transfer				<0.001				<0.001
Yes	18.0	11.5	22.0		18.2	9.0	19.7	
No	78.8	85.4	74.7		78.4	88.4	76.8	
Unknown	3.2	3.2	3.3		3.4	2.7	3.6	
Year				0.59				0.17
1998	25.4	26.0	25.1		22.0	25.6	21.4	
1999	23.7	21.5	25.1		22.2	24.2	21.9	
2000	25.5	25.2	25.7		26.0	25.6	26.1	
2001	25.3	27.4	24.0		29.8	24.6	30.6	
Procedure-specific variables								
Same admission PCI	2.8	3.3	2.4	<0.001	-	-	-	-
Concomitant valve procedure	10.6	9.5	11.3	<0.001	-	-	-	-
Internal mammary artery graft	16.8	13.4	18.8	<0.001	-	-	-	-
Multivessel PCI	-	-	-	-	14.5	14.8	12.6	<0.001

Unless noted otherwise, findings are expressed as percentages

Percentages may not sum to 100 due to rounding

* Hospital year groups refer to the number of hospitals that contributed data in each year of the NIS. A hospital participating in the NIS over the 3 year period would be considered to have contributed 3 hospital year groups to the analysis.

### Hospital procedure volume and mortality

Crude in-hospital mortality was 3.64% for patients undergoing CABG and 1.50% for patients undergoing PCI. In-hospital mortality rates were higher for patients treated in low volume hospitals compared with high volume hospitals for CABG (3.85% vs. 3.51%, P = 0.002) and PCI (1.96% vs. 1.43%, P < 0.001). Patients at low volume hospitals remained at increased risk of in-hospital mortality after multivariable adjustment for CABG (odds ratio [OR] 1.16, 95% confidence interval [CI] 1.10–1.24) and PCI (OR 1.12, 95% CI 1.05–1.20) compared with patients at high volume hospitals (Table 2).

**Table 2:** Patient mortality by hospital volume groups

	**Hospital CABG Volume**			
	**Overall**	**Low (<450)**	**High (≥450)**	**P**
Crude rates	3.6	3.9	3.5	0.002
Unadjusted odds ratio (95% CI)	-	1.10 (1.04–1.16)	1.00 [referent]	0.001
Adjusted odds ratio (95% CI)	-	1.16 (1.10–1.24)	1.00 [referent]	<0.001
	**Hospital PCI Volume**			
	**Overall**	**Low (<400)**	**High (≥400)**	**P**

Crude rates	1.5	2.0	1.43	<0.001
Unadjusted odds ratio (95% CI)	-	1.37 (1.28–1.49)	1.00 [referent]	<0.001
Adjusted odds ratio (95% CI)	-	1.12 (1.05–1.20)	1.00 [referent]	0.001

### Impact of hospital procedure volume minimums

Implementation of a hospital procedure volume minimum policy for cardiovascular procedures would require the transfer of an estimated 231,348 total patients each year, 143,687 patients for CABG, and 87,661 for PCI. A best-case estimate suggests the transfer of patients from low volume to high volume hospitals annually could have prevented 728 in-hospital deaths. The majority of annual deaths prevented by the transfer of patients to high volume hospitals were for patients undergoing CABG (619 deaths, 4.5% of all CABG deaths), with 109 deaths (1.2% of all PCI deaths) avoided among patients undergoing PCI. Adoption of a hospital volume minimum policy would thus require the transfer of 232 patients from low volume to high volume CABG hospitals to avert a single death, and 805 patients from low volume to high volume PCI hospitals to avert a single death (Table 3).

**Table 3:** Volume and mortality estimates

	**CABG**	**PCI**
**Current**		
Total procedures, n(%)	374,234	625,199
Performed at LVH, n(%)	143,687	87,661
Performed at HVH, n(%)	230,547	537,538
		
Total in-hospital deaths, n(%)	13,633	9,405
Performed at LVH, n(%)	5,535	1,719
Performed at HVH, n(%)	8,098	7,686
		
**Adopting volume minimum policy**		
Total procedures, n(%)	374,234	625,199
Performed at LVH, n(%)	0	0
Performed at HVH, n(%)	374,234	625,199
		
Total in-hospital deaths, n(%)	13,014	9,296
Performed at LVH, n(%)	0	0
Performed at HVH, n(%)	13,014	9,296
		
**Impact of adopting volume minimum policy**		
Procedures moved from LVH to HVH, n	143,687	87,661
		
Deaths averted, n	619	109
		
Number of procedures moved from LVH to HVH to avoid 1 death	232	805
		
Percent reduction in deaths	4.54%	1.16%

## Discussion

The nationwide implementation of a hospital volume minimum policy for cardiovascular procedures based on volume thresholds promoted by the Leapfrog Group [7] would have required the annual redistribution of more than 231,000 patients who underwent CABG or PCI at low volume hospitals between 1998 and 2001. At best, this redistribution would have resulted in approximately 728 fewer deaths annually, concentrated primarily among patients who underwent CABG (619 deaths). This suggests that previously reported mortality benefits associated with volume minimums for PCI may be overstated (547 deaths averted vs. 109) [7]. Differences between our estimates and previous ones are driven more by methodology than data. Whereas previous studies assumed that all patients moved from low- to high-volume centers would receive the same average mortality benefit, our methodology calculated the expected benefit for each patient at a low-volume center based on the volume of the center and the patient's comorbidities.

The potential to avert up to 728 deaths each year through the treatment of CABG and PCI patients at only high volume hospitals may be interpreted as sufficient evidence for the adoption of a procedure volume minimum policy. However, this benefit must also be considered in the context of the required transfer of over 231,000 patients each year from low volume to high volume hospitals. Because the average absolute incremental increase in mortality associated with treatment at a low volume hospital compared with a high volume hospital is generally small (<0.5%), particularly for patients undergoing PCI, a large number of patients would need to be treated at high volume hospitals (805 for PCI) in order to avert a single death. This number needed to treat is larger than that of most current cardiovascular drugs and therapies, suggesting only a modest benefit for any individual patient [16]. Moreover, the number of deaths attributable to treatment at low versus high volume hospitals represents only a small proportion of overall procedure mortality (4.5% of CABG deaths, 1.2% of PCI deaths). Recent studies also suggest substantial heterogeneity in CABG and PCI outcomes among hospitals, including low volume hospitals with better than predicted outcomes and high volume hospitals with worse than predicted outcomes [17,18]. As such, hospital volume may be both a modest and unreliable measure of any individual hospital's performance [19].

Policies regulating hospital procedure volume minimums may also have potential adverse consequences. Concentrating services among fewer providers may adversely affect access to procedures in many areas of the country [20]. Differences in patient characteristics between low and high volume hospitals in our analysis suggest the suspension of services at low volume hospitals may disproportionately affect minorities and patients with Medicaid insurance, groups with historically limited access to cardiovascular care. A reduction in the number of CABG and PCI providers may also result in higher prices for health care purchasers as provider competition is reduced. Adoption of volume thresholds may unwittingly motivate providers to treat patients with borderline indications in order to meet volume minimums. Patients may not support regionalization of procedures associated with hospital volume minimums if they prefer receiving care at local, low volume hospitals [21]. Each of these and other factors requires consideration prior to adopting any hospital volume minimum policy.

### Limitations

Although our analysis provides estimates of the mortality reductions that may be achieved through hospital volume minimums, we necessarily make several assumptions. First, we assumed that high volume hospitals achieved superior outcomes because of their higher volumes [22]. If high volume hospitals have better outcomes because of patient selection or factors other than hospital volume itself, the mortality benefit and number of lives saved reported by transferring patients from low volume hospitals may be overstated. Second, our analysis assumes that all patients can be shifted from low volume hospitals to high volume hospitals and accrue a hospital volume-associated mortality benefit. No study to date has tested this assertion, and its validity remains unknown [23]. Third, because it is impossible to know the exact volume of the high volume hospital to which a low volume hospital patient would be transferred, we assumed that all low volume hospital patients would receive the average benefit of being treated at a high volume hospital. Finally, our analysis assumes that redistributing patients across hospitals has no adverse consequences.

Our study has a number of limitations. First, the NIS database is based on administrative data, and may be susceptible to hospital-based variations in coding practices. Previous studies, however, have demonstrated that administrative databases contain sufficient information to evaluate hospital differences in procedure quality [24], and the NIS is a comprehensive, nationally representative, all-payer database that includes information on cardiovascular procedure use. Second, we evaluated in-hospital mortality and were unable to assess other outcomes, including procedural complications or post-discharge events. However, the Leapfrog Group's volume recommendations are predicated on a mortality reduction, not improvement on other outcomes [6,7]. Third, the NIS does not collect data concerning physician volume, and thus we could not assess the effect of operator volume. However, previous studies suggest hospital volume is associated with outcomes even after accounting for operator volume [13,25,26]. Learning by doing and/or (dis)economies of scale, which are not captured in this analysis, may also influence estimates of the impact of a hospital volume minimum policy. Finally, the NIS does not contain unique patient identifiers, and the possible inclusion of multiple patient admissions in our cohort may violate statistical assumptions of independence.

## Conclusion

Implementation of a hospital volume minimum policy for CABG and PCI based on the Leapfrog Group's Evidence Based Hospital Referral guidelines in effect until 2003 would have required the annual redistribution of over 231,000 patients from low volume hospitals to high volume hospitals between 1998 and 2001. This policy would have resulted in, at best, an estimated 728 fewer deaths annually, primarily among patients undergoing CABG. These estimates rely on the unproven assumption that simply directing all patients to high volume hospitals would eliminate the full differential in mortality between low and high volume hospitals. Further, given the uncertain feasibility and potential adverse consequences, requiring the movement of 232 patients for CABG or 805 patients for PCI between hospitals to avert a single death may not be an effective policy. These issues deserve further study before hospital procedure volume minimum policies for CABG and PCI are adopted more widely by purchasers.

## Competing interests

The authors declare that they have no competing interests.

## Authors' contributions

AJE and SSR were the principal authors of the manuscript. AJE was the principal data analyst. AJE, SSR, HMK and KGMV participated in study conception, interpretation, and drafting of the manuscript. AJE and KGMV were involved with data acquisition. All authors read and approved the final manuscript.

## Pre-publication history

The pre-publication history for this paper can be accessed here:


